# Performance Regulation of Thieno[3,2-b]benzothiophene π-Spacer-Based D-π-A Organic Dyes for Dye-Sensitized Solar Cell Applications: Insights From Computational Study

**DOI:** 10.3389/fchem.2018.00676

**Published:** 2019-01-29

**Authors:** Xiaoyin Xie, Zhi-Hai Liu, Fu-Quan Bai, Hong-Xing Zhang

**Affiliations:** ^1^International Joint Research Laboratory of Nano-Micro Architecture Chemistry, Institute of Theoretical Chemistry, Jilin University, Changchun, China; ^2^Department of Chemical Technology, Jilin Institute of Chemical Technology, Jilin, China; ^3^School of Opto-Electronic Information Science and Technology, Yantai University, Shandong, China

**Keywords:** organic dye, fused heterocyclic π-spacer, DSSC, DFT, charge transfer

## Abstract

Dye-sensitized solar cells (DSSCs) have been widely investigated; however, the development of promising dye sensitizers is still appealing. In this work, we perform a detailed theoretical search for high-efficiency D-π-A organic dyes using density functional theory and time-dependent density functional theory calculations. Specifically, we perform geometric optimization, and electronic structure and absorption spectra calculations for isolated dyes for two thieno[3,2-b]benzothiophene π-spacer-based D-π-A organic dyes SGT129 and SGT130, which show significant efficiency difference, before and after binding to a TiO_2_ semiconductor. The calculation results reveal that the coplanar configuration between the electron donor and the π-spacer can enhance electronic communication efficiently, thus facilitating intra-molecular charge transfer from the electron donor to the acceptor groups in SGT130. The absorption spectrum of SGT130 broadens and is red-shifted owing to the decreased bandgap. The higher light-harvesting efficiency, favorable intra-molecular charge transfer, larger shift of the conduction band edge in the TiO_2_ semiconductor, and slower charge recombination between the injected electrons in the TiO_2_ conduction band and the electrolyte explain the superior efficiency of SGT130 over that of SGT129. Using SGT130 as the reference dye, we further design four novel dyes **1**–**4** by modifying the π-spacer with electron-rich and electron-withdrawing moieties. Judging from the theoretical parameters influencing the short-circuit current and open-circuit voltage, we found that all dyes would perform better than SGT130 in terms of the favorable interfacial charge transfer (ICT) and light-harvesting efficiency, as well as the larger shift of the TiO_2_ conduction band edge. Our theoretical research is expected to provide valuable insights into the molecular modification of TBT-based D-π-A organic dyes for DSSC applications.

## Introduction

Depletion of fossil fuels and the resulting environmental issues have led to increased interest in the search for alternative energy sources. The solar cell, a device that converts solar energy into electricity, offers a valuable solution to this problem. Among various photovoltaic devices, dye-sensitized solar cells (DSSCs) are prominent owing to their obvious advantages of low cost, high power conversion efficiency, and easy fabrication relative to Si-based solar cells (O'Regan and Grätzel, 1991; Grätzel, 2001). DSSCs have the following four primary parts: dye sensitizer, TiO_2_ semiconductor, electrolyte, and counter electrode. Among them, the dye sensitizer is crucial in the operation of DSSCs as it is responsible for the capture of photons and generation of photo-excited electrons. The excited electrons are subsequently injected to the semiconductor conduction band, leaving the oxidized dye for subsequent reduction by an electrolyte.

The DSSC performance can be tuned by regulating the molecular structure of the dye sensitizer while keeping the other components unchanged. In the past decades, a large number of attempts have been made to develop novel dyes (Chen et al., 2011; Tseng et al., 2014; Wu et al., 2015; Mandal et al., 2016; Eom et al., 2017; Li P. et al., 2017b; Li Y. et al., 2017; Wang et al., 2017; Zhang et al., 2017; Dhar et al., 2018; Li et al., 2018a; Lu et al., 2018a,b; Wen et al., 2018; Xu et al., 2019) for DSSCs. Polypyridyl ruthenium complex dyes (e.g., N719; Chung et al., 2012) have been demonstrated to result in high device efficiency. However, ruthenium dyes are inappropriate for large-scale solar energy utilization applications because they are costly and cause environmental pollution. Continuous research efforts have been devoted to the development of low-cost, environment-friendly, and easily tuneable metal-free organic sensitizers (Zhang et al., 2012a; Li et al., 2014c,d; Li M. et al., 2015; Arbouch et al., 2017; Li P. et al., 2017a; Lu et al., 2018b). In addition, organic dyes have higher molar extinction coefficients and a broader absorption band in the long-wavelength region of the solar spectrum. However, the efficiency of organic dyes is relatively low compared with that of ruthenium dyes, but it can be improved by extensive structural modification owing to their geometrical flexibility.

Most organic dyes have a D-π-A structure, where D/A and π refer to the electron donor/acceptor and π-bridge groups, respectively. This type of structure facilitates intra-molecular charge transfer upon photo excitation with a conjugated linker acting as a bridge to enable the transfer of electrons generated by the donor group to the acceptor group. The electron acceptor grafts the dye onto the semiconductor surface; subsequently, the exited-state electrons from the dye sensitizer can be injected into the conduction band (CB) of TiO_2_ by appropriate energy alignment of the dye/semiconductor interface system. Generally, the following points must be considered when designing novel dyes: the highest occupied molecular orbital (HOMO) should be below the redox potential of I^−^/${\text{I}}_{3}^{-}$; the lowest unoccupied molecular orbital (LUMO) above the conduction band edge must facilitate dye regeneration and electron injection; the HOMO–LUMO gap must be small to maximize light-harvesting efficiency; and strong electronic coupling must exist between the LUMO of the dye and the CB of TiO_2_ to ensure fast interfacial charge transfer (ICT). All these factors have been discussed widely in previous works (Li W. et al., 2015; Lu et al., 2018b) and used as guidelines to screen potential candidates for dye sensitizers.

The π-bridge group is crucial in enhancing the performance of DSSCs because it can primarily affect the HOMO and LUMO energy levels, and thus, the absorption spectrum that is important for light harvesting. In addition, the planar conformation of the π-bridge group favors the electronic communication between the electron donor and acceptor groups, and facilitates intra-molecular charge transfer. A large number of π-bridge groups have been developed from experimental and theoretical viewpoints (Bouit et al., 2012; Zhang et al., 2012b, 2014; Cai et al., 2013; Gao et al., 2014; Eom et al., 2015). Recently, π-bridges based on fused heterocyclic moieties such as thieno[3,2-*b*]benzothiophene (TBT**)** have drawn significant attention, and their potential application as the core for functionalization has been demonstrated in the field of organic transistors and ferroelectric liquid crystals given their advantages such as planar conformation, asymmetric structure, and thermal stability (Košata et al., 2004). In addition, these π-bridges perform well as coupling mediators owing to their good intra-molecular charge transfer properties. However, the weak electron-donating ability of the TBT moiety owing to the presence of benzothiophene (BDT) limits extensive applications of the corresponding π-bridges. This problem can be solved organically by utilizing the electron-withdrawing ability of BDTs, which can enhance photo absorption and allow us to introduce the TBT moiety into the π-bridge group of D-π-A organic dyes.

**Figure 1:**
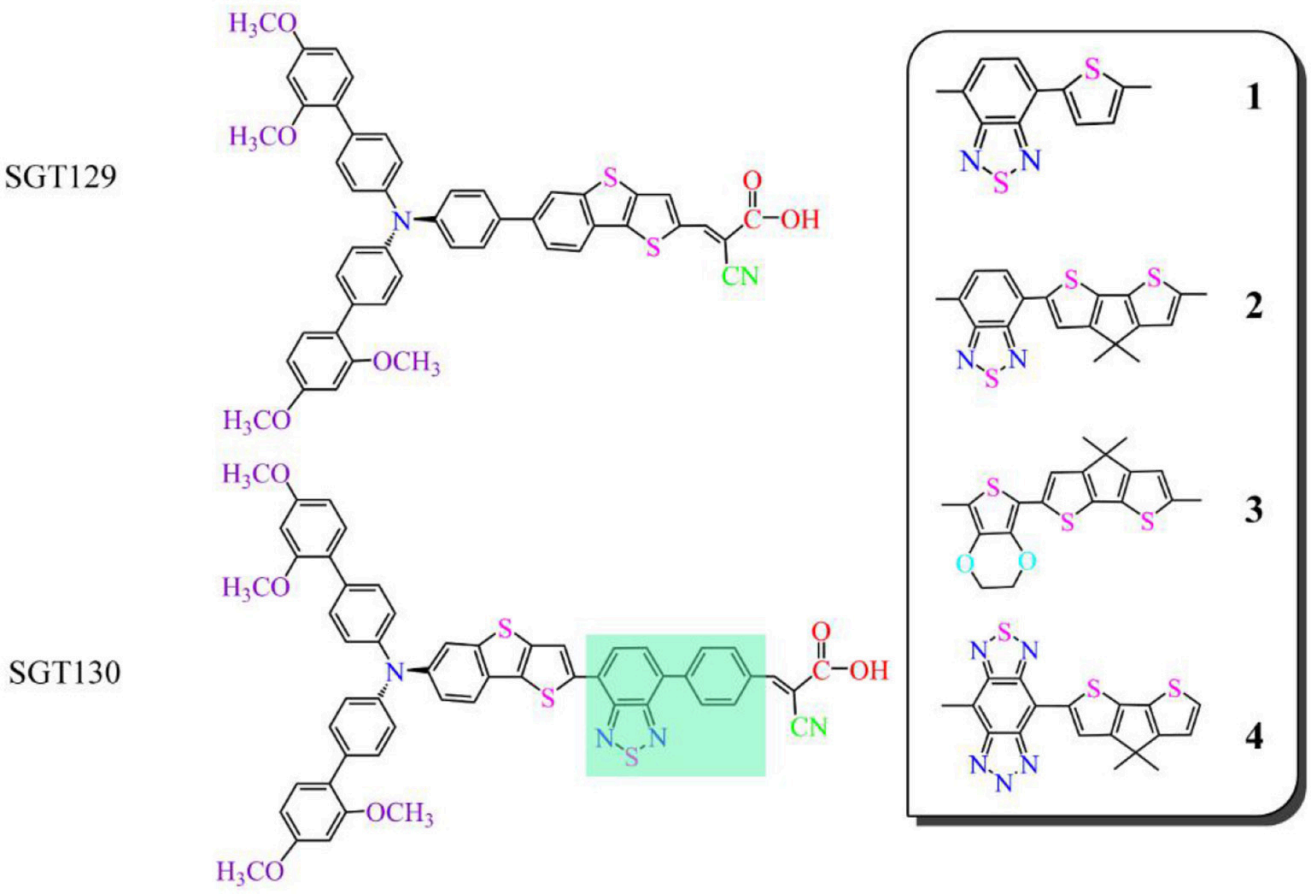
Sketch representation of the studied dyes.

Recently, Eom et al. reported a series of TBT-based organic dyes for DSSC applications (Eom et al., 2015). The representative dyes are SGT129 and SGT130, as illustrated in Figure 1; both dyes comprise a triphenylamine (TPA), TBT unit, and cyanoacrylic acid as the electron donor, π-bridge, and electron acceptor groups, respectively. The primary difference between these two dyes is that SGT129 contains a TBT π-bridge group, whereas in SGT130, the phenyl unit of the TPA donor is fused with the thiophene unit of the TBT group. According to experiments, the power conversion efficiency of SGT130 after the introduction of a benzothiadiazole-phenyl (BTD-P) unit as an auxiliary acceptor is twice that of SGT129. The reason for a large difference in efficiency between the dyes is intriguing given the small structural variation, and this has triggered research toward improving the performance of SGT130. Herein, we first rationalize the experimentally observed performance difference between SGT129 and SGT130 by density functional theory (DFT) calculations and time-dependent DFT (TDDFT) investigations. We calculate the geometrical structure, electronic structure, and optical properties of the isolated dyes, as well as the charge transfer at the dye/TiO_2_ interface, which is the basis of the photo-to-current conversion device. Subsequently, we designed four novel dyes based on SGT130 by substitution of the BTD-P unit with other frequently used electron-withdrawing groups. The calculation results indicated that the SGT130 dye has enhanced photocurrent and voltage compared to the SGT129 dye owing to its better optical absorption, efficient intra-molecular charge transfer, large TiO_2_ CB shift, and slow charge recombination between the injected electron in the semiconductor and electrolyte, which agreed with the experimental results. We further demonstrated that all the designed dyes would be potential high-efficiency dye sensitizers as they perform well on key parameters influencing the photovoltaic efficiency. The current theoretical research demonstrates the potential of fine tuning the TBT-based π-spacer to develop highly efficient D-π-A organic dyes, which is of particular importance for further optimization of DSSCs.

## Computational Details

### Theoretical Method

The performance of DSSCs can be characterized by the power conversion efficiency (PCE, η), and can be expressed as follows:

(1) $$\begin{array}{l} \text{η}={\text{J}}_{\text{sc}}^{*}{\text{V}}_{\text{oc}}^{*}ff/{\text{I}}_{\text{s}} \end{array}$$

Where *J*_*sc*_ is the short-circuit current density of DSSCs under the sunlight irradiation, *ff* is the filling factor, and *V*_*oc*_ is the open-circuit photovoltage under the same operation condition. *V*_*oc*_ can be determined by the following formula (Marinado et al., 2010):

(2) $$\begin{array}{l} {\text{V}}_{\text{oc}}=\frac{{\text{E}}_{\text{cb}}}{\text{q}}+\frac{\text{κT}}{\text{q}}\ln\left(\frac{{\text{n}}_{\text{c}}}{{\text{N}}_{\text{CB}}}\right)-\frac{{\text{E}}_{\text{redox}}}{\text{q}} \end{array}$$

Where *q* is the unit charge, *E*_*cb*_ is the CB edge of the TiO_2_ semiconductor, and *E*_*redox*_ is the redox potential of the ${\text{I}}_{3}^{-}$**/**I^−^ electrolyte; κT is the thermal energy under room temperature; N_CB_ is the effective state density of the TiO_2_ conduction band; *n*_*c*_ is the number of electrons in the conduction band. Obviously, from equation 2, *V*_*oc*_ depends primarily on the energy difference of *E*_*cb*_ and *E*_*redox*_; a larger shift in the TiO_2_ CB edge (ΔE_cb_) will produce a positive impact on the *V*_*oc*_. *J*_*sc*_ can be expressed as follows (Lu et al., 2017):

(3) $$\begin{array}{l} {\text{J}}_{\text{sc}}=\text{e}\int_{\text{λ}}\text{LHE}\left(\text{λ}\right){\text{φ}}_{\text{inject}}{\text{η}}_{\text{collect}}{\text{η}}_{\text{reg}}{\text{φ}}_{\text{ph}.\text{AM}1.5\text{G}}\text{dλ} \end{array}$$

Where LHE(λ) is the light-harvesting efficiency at a given wavelength, λ; φ_*inject*_ is the electron injection efficiency; η_*collect*_ is the charge collection efficiency; η_*reg*_ is the dye regeneration efficiency; φ_*ph*.*AM*1.5*G*_ is the photon flux under AM1.5 solar irradiation; LHE(λ) can be further determined by the following equation:

(4) $$\begin{array}{l} \text{LHE}\left(\text{λ}\right)=1-10^{-\text{ϵ}\left(\text{λ}\right)\text{bc}} \end{array}$$

Where ε(λ) is the molar absorption coefficient associated with the excited states, *b*, and *c* are constants that are determined by the DSSC device. The theoretical maximum photocurrent $J_{sc}^{\max}$ can be obtained if φ_*inject*_, η_*collect*_, and η_*reg*_ equal 1. $J_{sc}^{\max}$ reflects the maximum and idealobtainable photocurrent.

### Computational Methods

Optimizations and electronic structure calculations of free dyes were performed using the Gaussian09 program (Frisch et al., 2009) using the B3LYP/6-31G(d) (Becke, 1993) level of theory in the gas phase. The absorption spectra were simulated using the CAM-B3LYP/6-31G(d) (Yanai et al., 2004) in the tetrahydrofuran (THF) solvent. The solvent effect was included with the polarizable continuum model (PCM) (Cossi et al., 2002). To reduce the computational cost, the long alkyl chains of all dyes were replaced by methyl groups, which has been validated in previous works (Li et al., 2014b). The charge recombination between the injected electron and electrolyte was evaluated by calculating the binding energy of I_2_ to different binding sites of the dye. Hence, the LanL2DZ [6-31G(d)] base set for I (C, H, O, N, S) atom were used (Hay and Wadt, 1985). We used the M06-2X DFT functional (Zhao and Truhlar, 2008) for the optimization of the dye-I_2_ system as it provides good results for such weak interaction systems. The basis set superposition error (BSSE) of the binding energy for the dye-I_2_ interaction was considered by the counterpoise method (Boys and Bernardi, 1970). The analysis of the excited electron density difference between the ground state and excited state was performed using the Multiwfn program (Lu and Chen, 2012). To study the interaction of the dye and TiO_2_ surface, we prepared a stoichiometric (TiO_2_)_38_ cluster and exposed its (101) surface. The (TiO_2_)_38_ cluster model was applied successfully to model the dye-sensitized TiO_2_ system (Li et al., 2014c,d; Li W. et al., 2015). For the geometric optimization of the (TiO_2_)_38_ cluster and dye/TiO_2_ combined system, the plane wave DFT method in conjugation with the generalized gradient approximation (GGA) of Perdew–Burke–Ernzerhwas used, as implemented in the Vienna *ab initio* simulation package code (Kresse and Hafner, 1993, 1994). The description of valence electrons is based on a plane wave basis set with an energy cut-off of 400 eV, using the gamma-only k-point. Vacuum layers were added to the x, y, and z directions to ensure no boundary interaction.

## Results and Discussion

Figure 1 shows the structures of the investigated dyes. It has been widely accepted that the planar conformation of the π-bridge group ensures the good electronic communication between the electron donor and acceptor, as shown by the work of Li P. et al. (2017a): We can expect that the torsion angles between the electron donor and π-bridge in SGT129 is larger than those in SGT130. this is due to the steric hindrance of the adjacent phenyl units from the TPA donor and TBT π-spacer. The use of a fused-ring building block in SGT130 reduces the dihedral angle between the thiophene unit and the phenyl unit of the TPA donor, thereby maintaining good coplanarity and rigidity. The improved coplanarity improves the π-conjugation between the electron donor and acceptor via the π-bridge, thus benefitting the electron delocalization and further intra-molecular charge transfer, as reported in early theoretical works (Li et al., 2014d; Li P. et al., 2017b).

**Figure 2:**
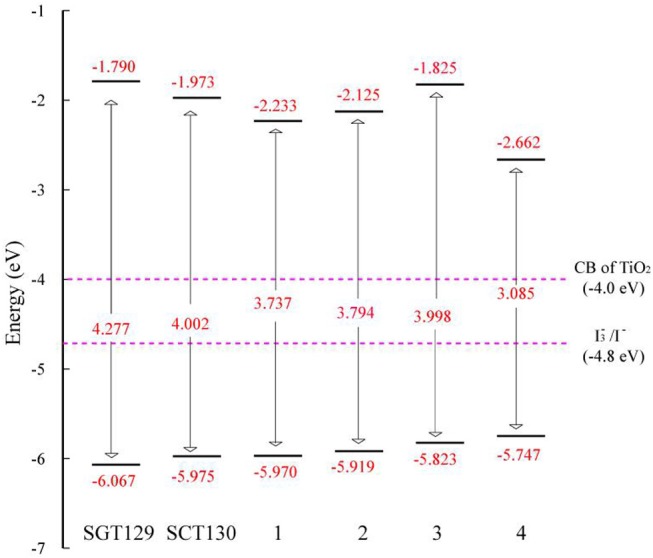
Calculated HOMO-LUMO energy levels and HOMO-LUMO energy gaps for all dyes.

**Figure 3:**
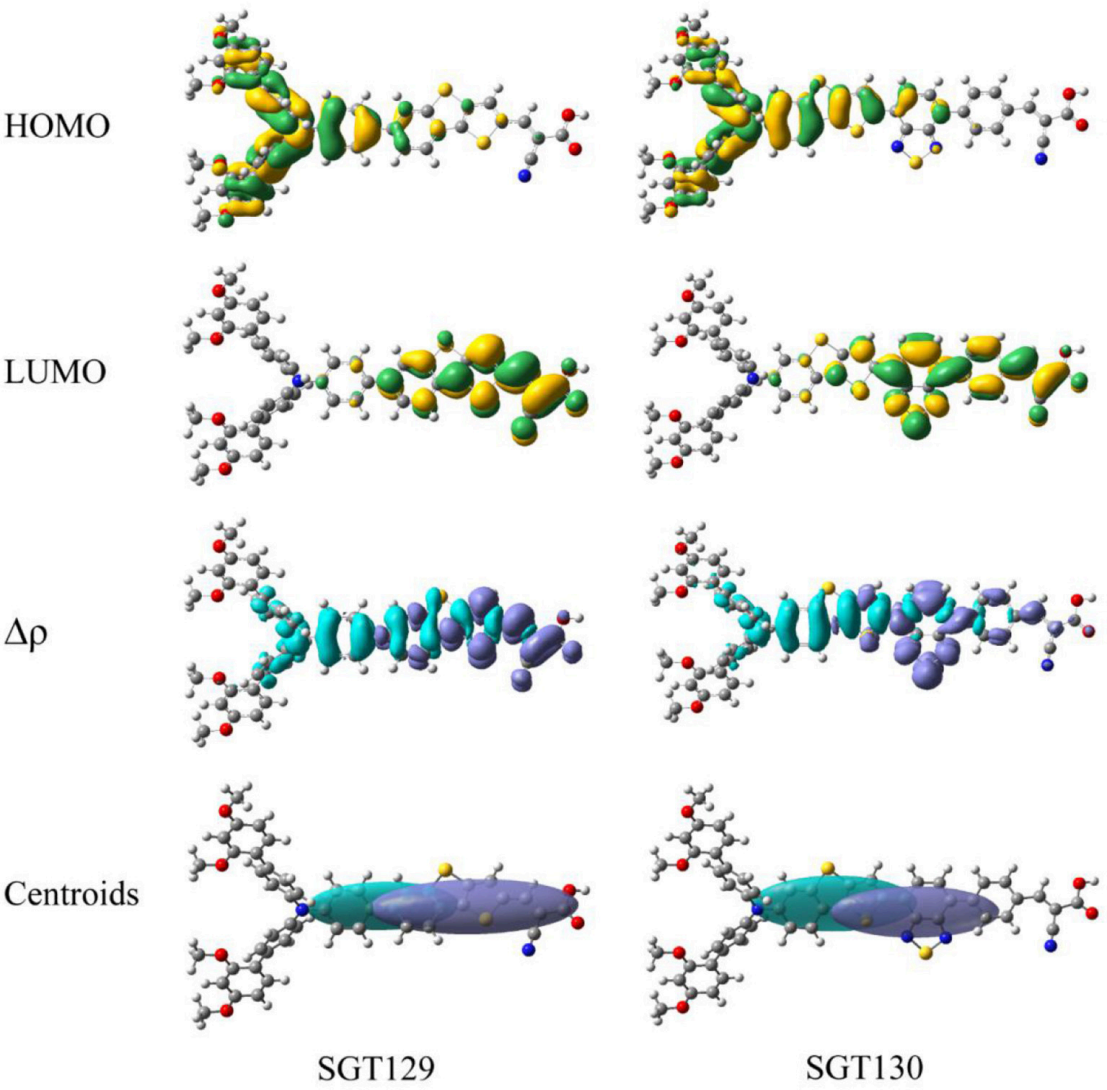
Computed HOMO and LUMO distributions, charge density difference(Δρ) between excited state and ground state, and centroids of charge density decreasing/increasing for **SGT129** and **SGT130**. The purple (blue) color indicates a charge density increasing (decreasing).

The intra-molecular charge transfer is closely related to the frontier molecular orbital energy levels and its distribution. Figure 2 presents the HOMO and LUMO energy levels and HOMO–LUMO energy gap calculated at the B3LYP/6-31G(d) level of theory. As shown, the HOMO level of SGT129 and SGT130 are located at −6.067 and −5.975 eV, respectively, and the HOMO of SGT130 is slightly upshifted by 0.1 eV. The Calculated LUMOs are −1.790 and −1.973 eV for SGT129 and SGT130, respectively, and a larger upshift of 0.163 eV is observed for the LUMO levels of SGT130. Both dyes exhibit HOMOs that are lower than the redox potential of I^−^/${\text{I}}_{3}^{-}$, and LUMOs that are higher than the conduction band edge of TiO_2_, thus guaranteeing the energetically favorable dye regeneration and electron injection. Compared with the SGT129 dye, the introduction of the BTD-P unit destabilizes both HOMO and LUMO levels, leading to the reduction in the HOMO–LUMO gap. The smaller HOMO–LUMO gap favors the harvest of more sunlight in the long-wavelength region. Figure 3 shows the charge density distribution of HOMO and LUMO of both dyes. For SGT130, the HOMO is primarily localized on the electron donor and TBT moiety, and LUMO is primarily distributed on the cyanoacrylic acid and adjacent π-spacer group. Both HOMO and LUMO exhibit large overlaps on the π-bridge group, thus creating favorable spatial distributions for the intra-molecular charge transfer process. It is interesting that the HOMO of SGT129 dye contributes less from the TBT moiety; this is due to the less planar structure between the TPA donor and TBT unit that is unfavorable for the electronic communication and intra-molecular charge transfer process. The intra-molecular charge transfer process can be visualized clearly from the charge density difference between the excited state and ground state, as shown in Figure 3. The blue (purple) area refers to the charge density decreasing (increasing) regions. For SGT129, most of the charge density decreasing regions occur at the electron donor, whereas the charge density increasing region occurs at the electron acceptor and TBT moiety. Using a fused-ring building block and the introduction of the BTP-P moiety render more decrease in charge density and increasing regions centered at the π-spacer. The analysis of two centroids C^+^/C^−^ corresponds to the increasing charge density and coincides with this observation.

**Figure 4:**
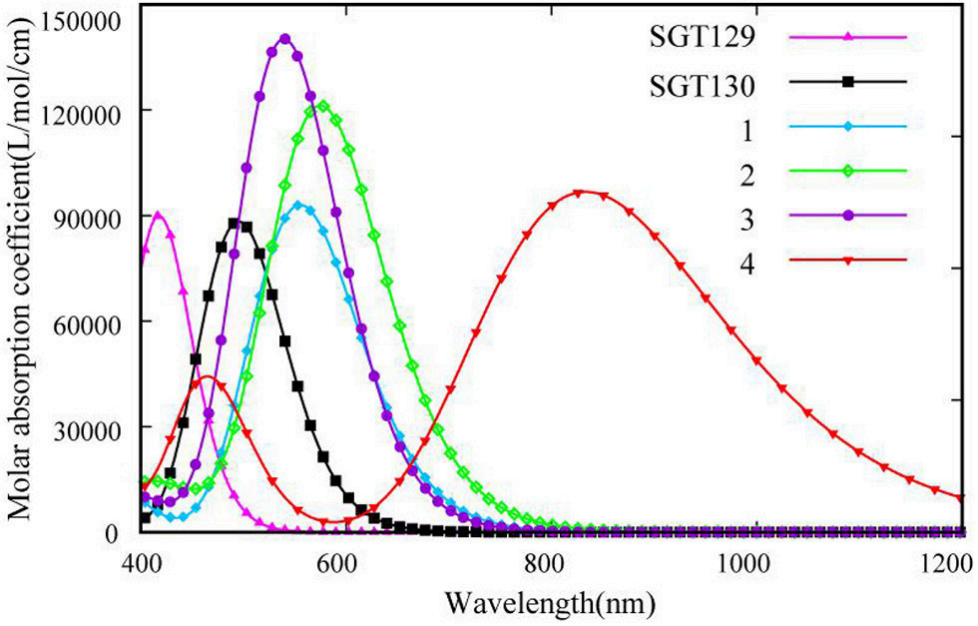
Simulated absorption spectra for all dyes at the PCM/TD-CAM-B3LYP/6-31G(d) level of theory.

**Figure 5:**
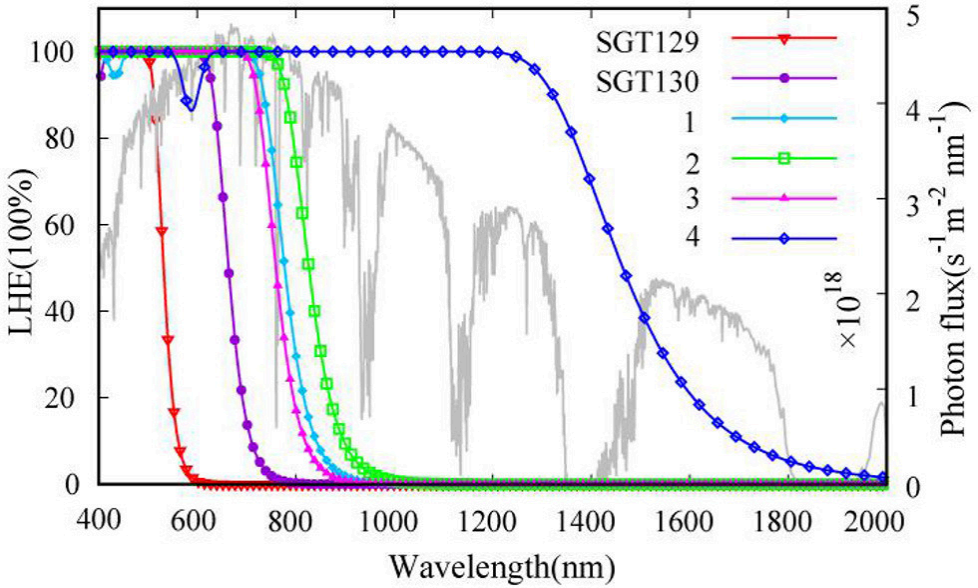
The sun light irradiation spectrum at AM1.5 (gray line) and calculated wavelength-dependent light-harvesting efficiency curves for all dyes.

We next evaluate the optical property of SGT129 and SGT130. It is well-known that the absorption spectrum of the dye sensitizer should encompass most of the solar spectrum to maximize sunlight harvesting. We simulated the absorption spectra at the PCM/TD-CAM-B3LYP/6-31G(d) level of theory in the THF solution, as presented in Figure 4. The excitation energy, oscillator strength, and electronic transitions are summarized in Table 1. As shown, all dyes comprise a major absorption band in the long wavelength region. The simulated absorption peaks for SGT129 and SGT130 are located at 417 and 494 nm, respectively, which agree with the experimental values of 426 and 514 nm, respectively. The absorption peak red shifted in SGT130 because of the decreased HOMO–LUMO gap; this can be further attributed to the better coplanarity between the electron donor and TBT unit, and the introduction of the electron-withdrawing BTD-P moiety. In addition, the relative trend on the intensity of the major absorption band agrees with the experimental observation (Eom et al., 2015). Therefore, the accuracy of the PCM/TD-CAM-B3LYP/6-31G(d) level of theory for the simulation of the optical properties of the investigated dyes can be validated. For both dyes, the lowest-lying excitation that dominates the primary absorption band can be attributed to the intra-molecular charge transfer based on the analysis above of the HOMO and LUMO charge density distributions. SGT130 exhibits the stronger light absorption ability owing to the red-shifted and broadened absorption spectrum that is necessary to produce a large photocurrent response. We also calculated the theoretical maximum photocurrent, $J_{sc}^{\max}$, using the theoretical methodology described in the Method section. The LHE(λ) curves are presented in Figure 5, and the calculated $J_{sc}^{\max}$ are listed in Table 1. The simulated $J_{sc}^{\max}$ are ~6.98 and 14.52 mA cm^−2^ for SGT129 and SGT130, respectively, which agree with the experimental trend (Eom et al., 2015).

**Table 1:** Calculated lowest-lying excitation energy, oscillator strength (*f*), charge transfer parameters, TiO_2_CB shift (ΔE_cb_), theoretical maximum photocurrent ($J_{sc}^{\max}$), interface charge transfer time (τ) of all dyes.

**Dye**	***ΔE_***cal***_* (eV)**	**λ_cal_ (nm)**	***F***	***dCT* (Å)**	***qCT* (*e*)**	***H* (Å)**	***t* (Å)**	**Δ*Ecb* (eV)**	**${\text{J}}_{\text{s}\text{c}}^{\text{m}a\text{x}}$ (mA cm^**−2**^)**	**τ (ps)**
SGT129	2.9688	417	1.6663	5.658	0.684	6.102	−0.444	0.159	6.98	0.037
SGT130	2.5050	494	1.6373	5.269	0.701	6.058	−0.789	0.198	14.52	0.125
1	2.2344	554	1.7246	4.971	0.665	6.194	−1.223	0.253	20.96	0.047
2	2.1582	574	2.2472	2.768	0.583	6.697	−3.929	0.292	23.63	0.027
3	2.2999	539	2.5987	4.208	0.576	6.227	−2.019	0.329	20.05	0.028
4	1.4891	832	1.7935	1.834	0.535	6.037	−4.203	0.363	42.97	0.154

To evaluate the extent of intra-molecular charge transfer quantitatively, we calculated the charge transfer parameters for the investigated dyes, such as *d*_*CT*_, *q*_*CT*_, *H*, and *t*, as listed in Table 1. *q*_*CT*_ represents the amount of the transferred charge. *d*_*CT*_ represents the distance of charge transfer; H represents the space distance between the centers of gravity of the density depletion/enhancement; *t* represents the degree of overlap between the charge density increasing/decreasing region. More details regarding these parameters can be found elsewhere (Le Bahers et al., 2011; Li et al., 2014d). It is shown that all the calculated t are smaller than 1.7 Å, which serve as the threshold of the through-space transition, as previously defined in literature(Ciofini et al., 2012; Jacquemin et al., 2012), indicating that both dyes exhibit no through-space transition character. The charge transfer distance is ~5.658 Å for SGT129 and 5.269 Å for SGT130. This difference can be rationalized through the analysis of charge density difference between the excited state and ground state, and the centroid of the charge density increasing and decreasing areas, as shown in Figure 3. The intra-molecular charge transfer in SGT130 is relatively more efficient as it contains the larger amount of transferred electron, 0.701 *e*, considerable length of charge transfer, 5.269 Å, and smaller *t*.

**Figure 6:**
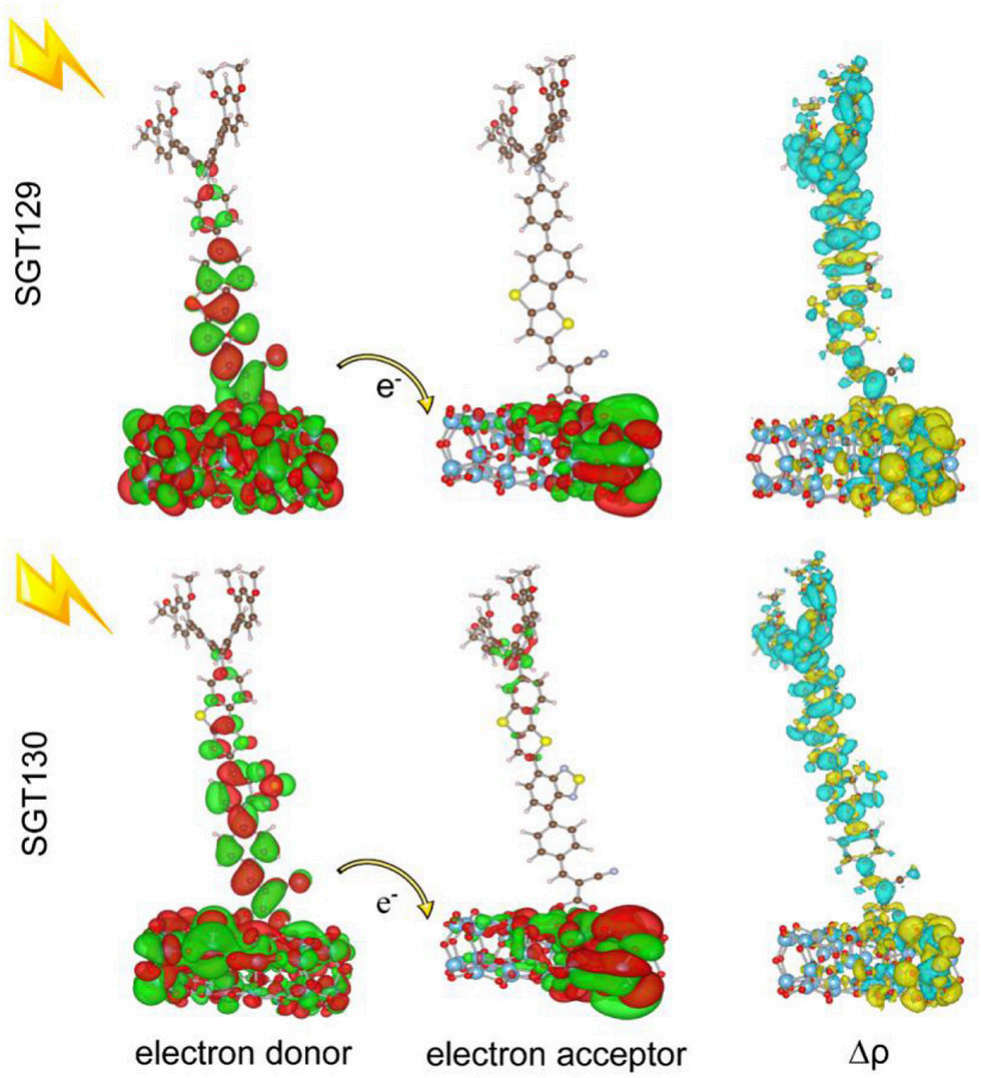
The charge density difference and electron donor and acceptor state responsible for interface charge transfer of dye-TiO_2_ systems.

The dye/TiO_2_interaction is important in determining the interface electron injection rate and thus the device performance. We adopted the (TiO_2_)_38_ cluster to model the surface structure of TiO_2_. The bidentate binding mode was used as the binding mode for the dye adsorption onto the (TiO_2_)_38_ cluster. This binding mode is the most stable form for the adsorption of dyes with cyanoacrylic acid acceptors (Li et al., 2014b). The (TiO_2_)_38_ cluster model has been widely used to investigate the dye/TiO_2_ interaction with smaller computation cost. Also, (TiI_2_)_38_ model has been demonstrated to nicely reproduce the bandgap of bulk TiO_2_ (Lundqvist et al., 2006). The interface charge transfer depends on the electronic coupling between the dye excited state and TiO_2_ CB state. To understand the mechanism of electron injection, we computed the electronic structure of the dye/TiO_2_ system, and present the electron donor and acceptor states together with the charge density difference between the excited state and ground state in Figure 6. Generally, the strength of electronic coupling is reflected by the overlap between the electron donor and acceptor states, as manifested by Fermi's golden rule. For SGT130, the electron donor group is distributed over the dye/TiO_2_ interface, and electron acceptor orbitals are localized on the TiO_2_ cluster. This is favorable for the interaction of electron donor and acceptor states, and thus the fast electron injection from dye excited state to TiO_2_ CB state. Inspection of the excited state charge density difference reveals that most of the electron decreasing and increasing regions are located at the dye/TiO_2_ interface, which is consistent with the analysis of the electron donor and acceptor states. Overall, the distribution of the electron donor and acceptor states creates a favorable overlap between the two states, and accelerates the interface charge transfer process.

**Figure 7:**
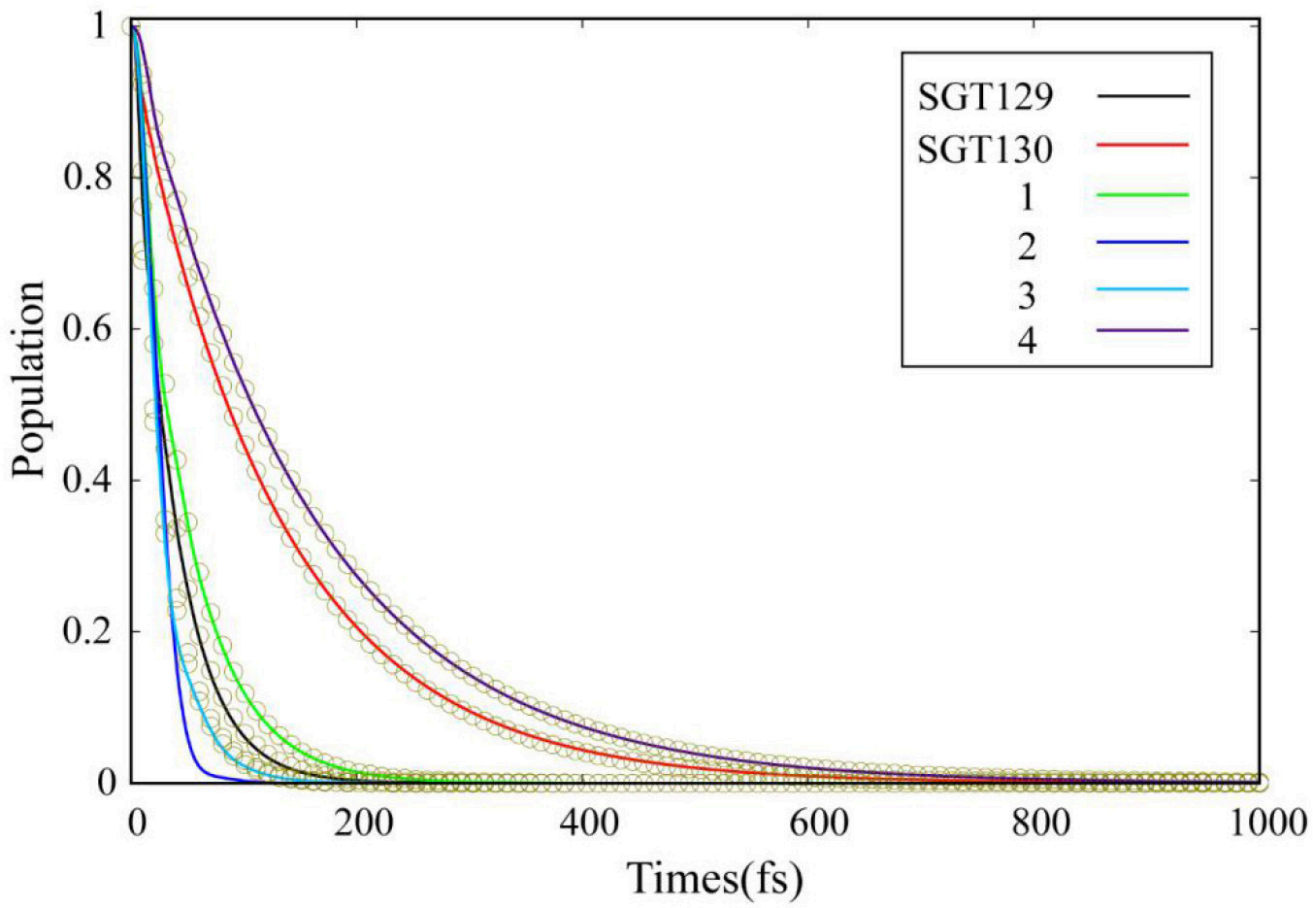
Time evolution of population starting from the photoexcited initial state.

Based on the optimized interface structure, we performed a quantum dynamics simulation of the interface charge transfer at the dye/TiO_2_ interface using the semi-empirical extended Hückel theory (EHT) combined with mean-field Ehrenfest dynamics. The electronic structure calculation is obtained using a tight binding EHT method. EHT method is able to provide the accurate results regarding the band structure and chemical bond with negligible computational costs due to the use of transferrable parameters compared to the *ab initio* method with the use of plane wave basis. EHT predicates well the properties of band and atomic bonding (Rego and Batista, 2003). The quantum dynamics method based on EHT and Ehrenfest methodology was developed by Rego et al. (Rego and Batista, 2003; Hoff et al., 2012; Monti et al., 2015; Torres et al., 2015) and has been applied successfully to investigate the interface charge transfer process in a number of systems (Li et al., 2014a,b; He et al., 2017; Lu et al., 2018a), with satisfied results achieved. More details regarding the validation of this methodology can be found elsewhere (Rego and Batista, 2003). During the simulation, the LUMO was chosen as the initial state for the propagation of the photo-excited electronic state, as the HOMO → LUMO transition represents the most probable photo excitation in these dyes. Figure 7 shows the time evolution of the population for the photo-excited initial state. P(t) is the time-evolved survival probability. We performed an exponential fitting, f(t) = exp(–t/τ), to obtain the electron injection time. The simulation results indicate that all dyes exhibit a fast timescale for the population decay compared to the nanoseconds charge recombination process (Li et al., 2018c). The fast electron injection process typically induces suppressed charge recombination and improves the photovoltaic efficiency (Li et al., 2018b,c,d,e).

**Figure 8:**
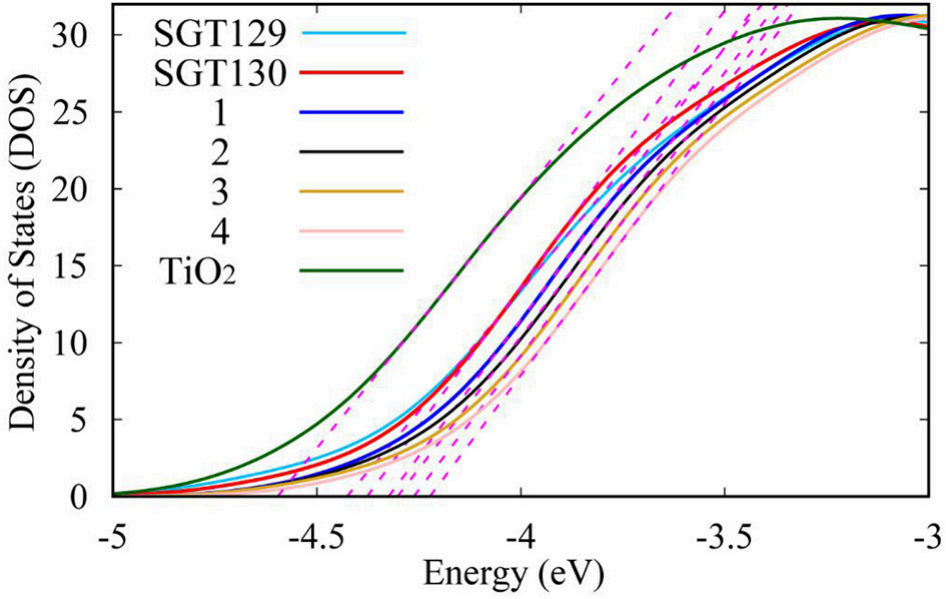
Illustration of the determination of TiO_2_ E_cb_ based on the partial density of states of (TiO_2_)_38_ cluster.

**Figure 9:**
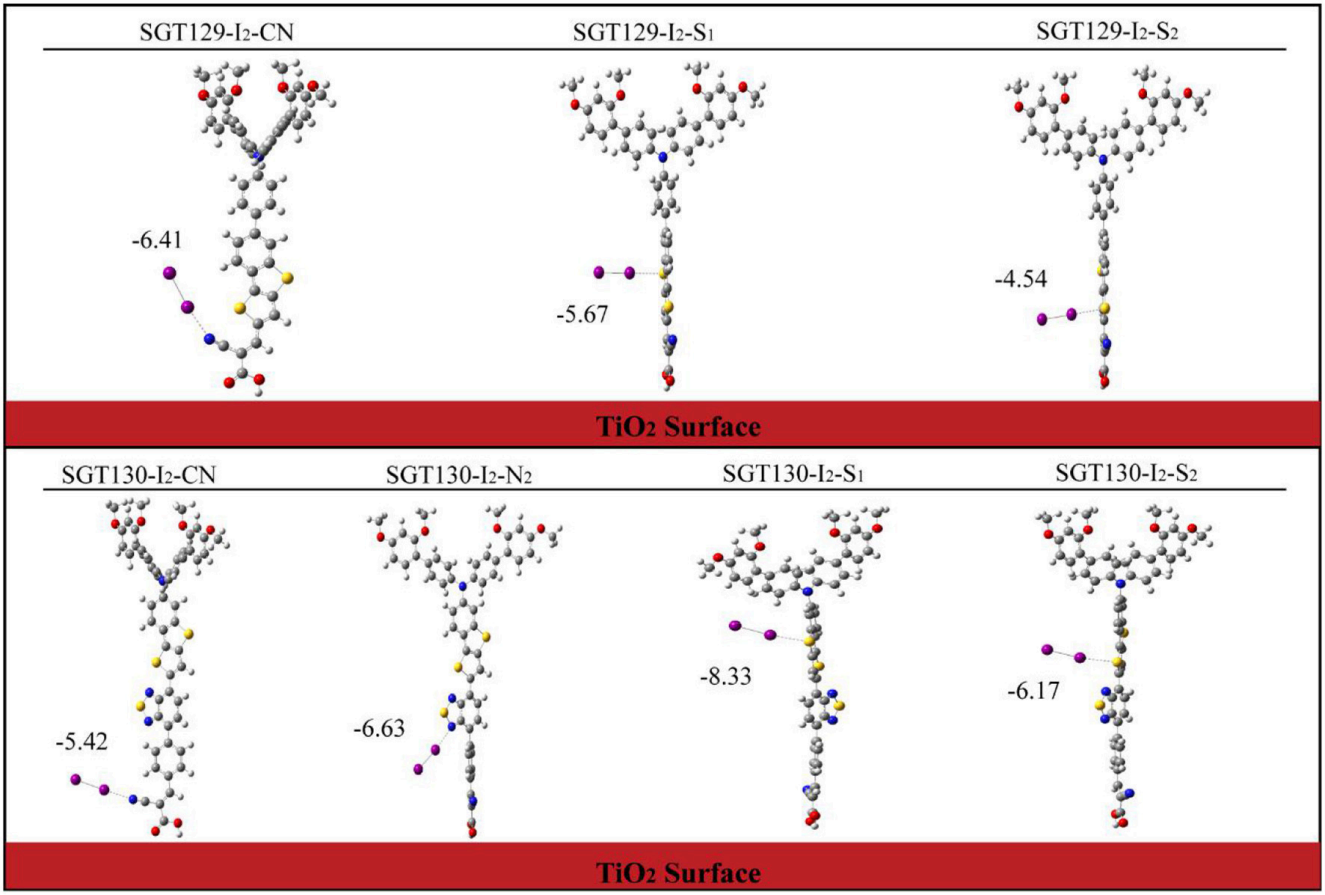
The optimized structures and binding energies of dye-I_2_ systems. Unit in kcal/mol.

Next, we will discuss the factors influencing *V*_*oc*_. As discussed in the Method section, *V*_*oc*_ primarily depends on the TiO_2_E_cb_ that changes upon the adsorption of different dyes. A larger shift in TiO_2_ E_cb_(ΔE_cb_) typically induces a larger *V*_*oc*_. To obtain the TiO_2_E_cb_, we computed the partial state density (pDOS) of the dye/TiO_2_ combined system using the B3LYP/6-31G(d) level of theory. Subsequently, a linear fitting of the low energy range of the pDOS from −5 to −3 eV was performed, and the intercept of the fitting line with the energy axis was chosen as the E_cb_ (see Figure 8). The computed ΔE_cb_ are listed in Table 1. As shown, SGT130 exhibits the larger ΔE_cb_; therefore, this dye would demonstrate a better *V*_*oc*_. In addition to the shift in TiO_2_E_cb_, *V*_*oc*_ was also subjected to the influence of the recombination between the injected electron in the TiO_2_ CB and electrolyte. In order to model this process, we consider the dye-I_2_ combined structure in which I_2_ binds to the possible binding sites of dyes, such as electron rich group, -CN, S, O, etc. Noted that we only consider the I_2_, previous simulations shown that the use of I_2_ for dye-electrolyte combined structure is more realistic and simplifies out simulation, whereas the overall conclusion is not changed. We present the optimized dye-I_2_ structure together with the binding energy in Figure 9. Generally, the I_2_ molecule must be placed far away from the TiO_2_ surface to minimize the recombination between the injected electrons in TiO_2_ and the electrolyte. The higher the binding site of I_2_, the less probable is the charge recombination. As shown for SGT129, the dye-I_2_-CN site possesses the largest binding energies of ~-6.41 kcal/mol than other sites. For SGT130, the most favorable binding site is the S1 atom of the TBT moiety with a binding energy equal to −8.33 kcal/mol. Therefore, for SGT130, the largest iodine concentration should occur around the S1atom, which might result in a slower charge recombination than SGT129. Overall, the larger the ΔE_cb_ and the slower the rationalized charge recombination, the better is the *V*_*oc*_ performance observed experimentally.

In this section, we focus on the further optimization of SGT130 dye. Structures of the designed dyes are shown in Figure 1. The new dyes were designed by replacing the BTD-P moiety with electron-rich moieties, e.g., thiophene, 4H-cyclopenta[2,1-b:3,4-b′]dithiophene (CPDT), and 3,4-ethylenedioxythiophene (EDOT), or electron-withdrawing moieties, e.g., BTD derivatives. First, it is noteworthy that all the HOMO and LUMO levels satisfied the requirement as an efficient dye: HOMO levels are below the redox potential of ${\text{I}}_{3}^{-}$/I^−^ and the LUMO levels above the TiO_2_ CB, see Figure 2. The HOMO–LUMO energy gap decreases in the order of **3** (3.998 eV) <**2** (3.794 eV) <**1** (3.737 eV) <**4** (3.085 eV). The changes in the HOMO and LUMO levels depend on the ability in donating and accepting electrons. For dye **3**, the introduction of electron-rich EDOT and CPDT moieties pushes the LUMO level up, thus enlarging the electron injection driving force. For dye **4**, the replacement of EDOT with an electron withdrawing moiety destabilizes the LUMO level, decreases the HOMO–LUMO energy gap, and thus favors sunlight harvesting. All the designed dyes exhibit a larger theoretical maximum photocurrent than the reference dye, SGT130. In particular, dye **4** possesses the largest $J_{sc}^{\max}$. This is because the designed dyes comprise either red-shifted absorption bands or larger absorption coefficients. The quantum dynamics simulation of the interface charge transfer suggests that dyes **1**–**3** exhibit the faster electron injection process, and that dye **4** exhibits the comparable timescale of electron injection as compared with SGT130. A fast electron injection is required to compete with other unfavorable processes, and to avoid energy and charge losses. All the designed dyes exhibit a larger Δ*E*_*cb*_ than SGT130, an indication of better *V*_*oc*_ performance. Based on the analysis above, we conclude that all the designed dyes exhibit better photocurrent and voltage performance than the SGT130 dye.

## Conclusions

We theoretically investigated and designed a series of TBT moiety-based D-π-A organic dyes for DSSC applications. DFT and TDDFT methods were used to perform the geometric optimization, and electronic structure and absorption spectra calculations for all dyes before and after binding to the TiO_2_ surface. The key parameters influencing *J*_*sc*_ and *V*_*oc*_ were discussed. The calculation results revealed that the coplanar configuration between the donor and π-spacer enhanced electronic communication, thus benefitting the intra-molecular charge transfer from the electron donor to the acceptor via the π-spacer. The HOMO and LUMO of all dyes were lower than the I^−^/${\text{I}}_{3}^{-}$ redox potential, and were higher than the conduction band of TiO_2_. Thus, the dye regeneration and electron injection process will be efficient. The absorption spectrum of SGT130 broadened and redshifted owing to the decrease in the HOMO–LUMO energy gap. Consequently, the attainable photocurrent response increased. SGT130 dye exhibited a larger Δ*E*_*cb*_ and slower charge recombination between the injected electron in the TiO_2_ CB and electrolyte, thus contributing to the better *V*_*oc*_ performance. We conclude that SGT130dye demonstrated a higher efficiency than SGT129, which agreed with the experiments. With SGT130 as the reference dye, we further designed four novel dyes by modifying the π-spacer with electron-rich and electron-withdrawing moieties. According to the theoretical parameters influencing the *J*_*sc*_ and *V*_*oc*_, all dyes are promising in challenging the efficiency of SGT130 because they performed well on the light-harvesting efficiency,$J_{sc}^{\max}$, Δ*E*_*cb*_, and interface charge transfer rate. Our theoretical research is expected to provide valuable insights into the further optimizations of D-π-A organic dyes for applications in DSSCs.

## Author Contributions

XX: design of the work data collection; Z-HL: data analysis and interpretation; F-QB: drafting the article; H-XZ: critical revision of the article.

### Conflict of Interest Statement

The authors declare that the research was conducted in the absence of any commercial or financial relationships that could be construed as a potential conflict of interest.
